# Supplementary material for: Interdiffusion and Intermetallic Compounds at Al/Cu Interfaces in Al-50vol.%Cu Composite Prepared by Solid-State Sintering
Source: Materials (Basel). 2021 Jul 31;14(15):4307. doi: 10.3390/ma14154307 (PMC8347343; doi:10.3390/ma14154307)
Supplement: Supplementary file 1 [file materials-14-04307-s001.zip › materials-1277547-supplementary.pdf]

materials

MDPI

Supplementary Material

Interdiffusion and Intermetallic Compounds at Al/Cu Interfaces in Al-50vol.%Cu Composite Prepared by Solid-State Sintering

Dasom Kim [1], Kyungju Kim [2] and Hansang Kwon [1,3,*]

[1] Department of Materials System Engineering, Pukyong National University, 48547 Busan, Korea; dasom.kim@f.mbox.nagoya-u.ac.jp
[2] The Industrial Science Technology Research Center, Pukyong National University, 48547 Busan, Korea; ngm13@ngm.re.kr
[3] Department of R&D, Next Generation Materials Co., Ltd., 48547 Busan, Korea
* Correspondence: kwon13@pknu.ac.kr

Citation: Kim, D.; Kim, K.; Kwon, H. Interdiffusion and Intermetallic Compounds at Al/Cu Interfaces in Al-50vol.%Cu Composite Prepared by Solid-State Sintering. *Materials* **2021**, *14*, 4307. https://doi.org/10.3390/ma14154307

Publisher's Note: MDPI stays neutral with regard to jurisdictional claims in published maps and institutional affiliations.

Copyright: © 2021 by the authors. Licensee MDPI, Basel, Switzerland. This article is an open access article distributed under the terms and conditions of the Creative Commons Attribution (CC BY) license (http://creativecommons.org/licenses/by/4.0/).

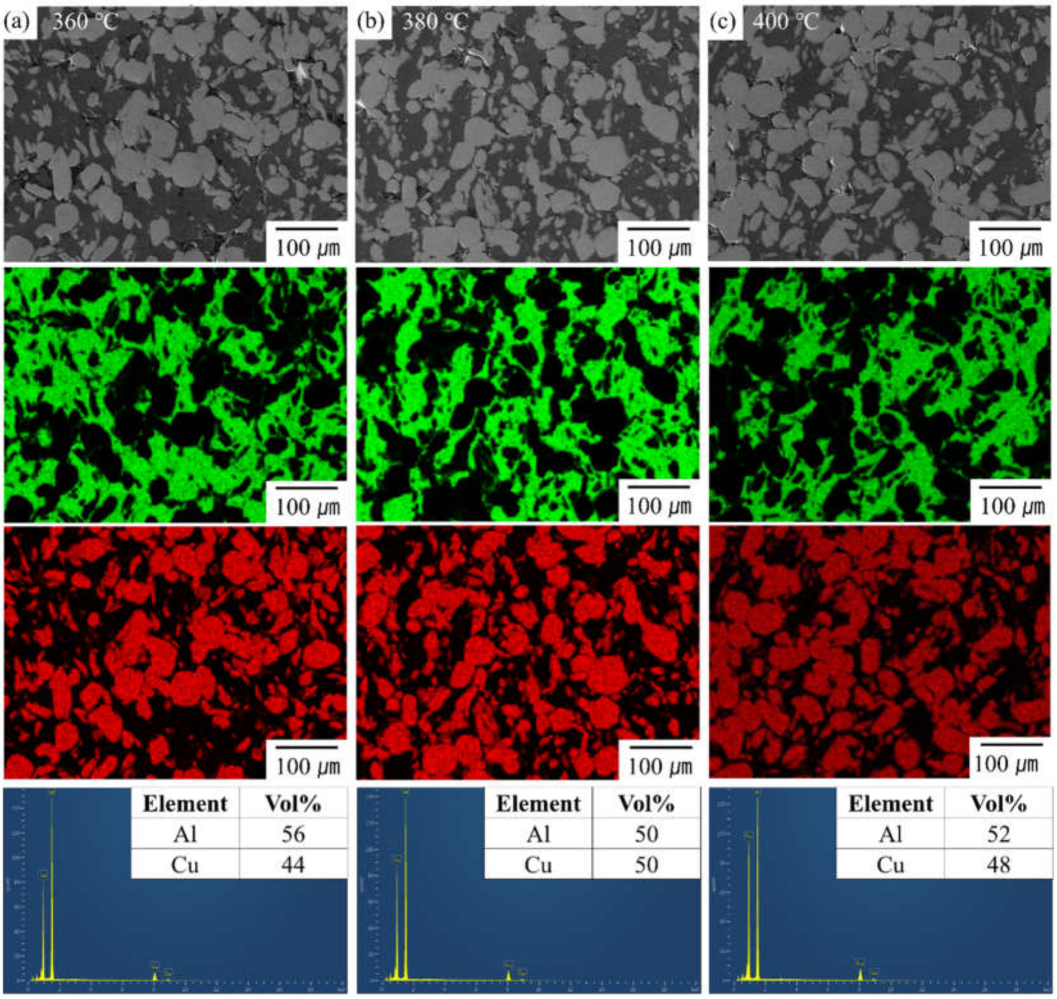

Figure S1. FE-SEM image and EDS mapping result with Al and Cu element of A-50vol.%Cu composites sintered at (**a**) 360 °C, (**b**) 380 °C and (**c**) 400 °C.
